# Capsular plication in the non-deformity hip: impact on post-operative joint stability

**DOI:** 10.1186/s40634-019-0172-x

**Published:** 2019-01-29

**Authors:** Etienne L. Belzile, Mathieu Hébert, Nicolas Janelle, Benoit Lechasseur, Yoann Dessery, Olufemi R. Ayeni, Philippe Corbeil

**Affiliations:** 1 CHU de Quebec-Université Laval, 11 cote du Palais, Quebec city, QC Canada; 2 Department of Surgery, Division of Orthopaedic Surgery, Faculty of Medicine, Université Laval, 1401 18e rue, Quebec city, QC G1J 1Z4 Canada; 3 Department of Kinesiology, Faculty of Medicine, Université Laval, Quebec city, QC, Canada; 4 Unité de recherche sur le vieillissement, Centre de recherche FRSQ du CHA universitaire de Québec, Quebec city, QC, Canada; 5 Division of Orthopaedic Surgery, McMaster University, Hamilton, Ontario Canada

**Keywords:** Hip arthroscopy, Capsulotomy, Hip instability, Capsular plication

## Abstract

**Purpose and hypothesis:**

The aim of this study was to evaluate the hip joint range of motion after different capsular plication. The study hypothesis proposed that capsular plication after hip arthroscopy may reduce hip external rotation and thus prevent the hip joint instability created by arthroscopic capsulotomies.

**Methods:**

Six fresh frozen human cadavers were studied in the intact state (5 males, 1 females) for a total of 12 non-deformity hips tested. They were fixed to the operating room table using a custom-made apparatus. Three Steinman pins were inserted, the first into ASIS, a parallel pin into the distal femur proximal to inter-epicondylar axis and the third pin into the lateral epicondyle. Simulation of arthroscopic capsulotomies was done progressively with simulation of three capsular plication techniques. The first plication technique consisted of a primary plication shift of the antero-lateral capsule. The distal-medial arm of the iliofemoral ligament was shifted toward the proximal-lateral arm. The second plication technique consisted in adding a longitudinal arm to the capsulotomy, between the lateral arm and the medial arm of the iliofemoral ligament, to create a T-shaped capsulotomy. The resulting two triangular capsular flaps were overlaid onto each other by approximately 5 mm, plicated fully and tighly sutured in a double-breast manner. The third plication technique, called redrapping, consisted in excising the inferior capsular triangular flap (previously made in the second technique), and suturing the latero-anterior superior capsular flap to the medial arm of the iliofemoral ligament, superimposing the capsular edges for closure. External rotation of the hip at 0°, 15° and 30° of flexion were obtained after the capsulotomy and each capsular plication technique to quantify the increase in hip stability after plication. Data were assessed using a two-way repeated measure analysis of variance (ANOVAs) and Student’s T-test when necessary to determine if the change in external rotation was significantly different.

**Results:**

After capsulotomy, external rotation averaged 26.3°, 29.1° and 31.1° at 0°, 15° and 30° of flexion. With the primary plication shift, external rotation averaged 24.9°, 30.3° and 34.0°. With the two-triangle technique, external rotation averaged 26.1°, 31.9° and 33.3°. With the re-draping technique, external rotation averaged 25.8°, 30.9° and 32.0°. A significant relationship was found between «Plication Technique» and «Angle of flexion» factors for the measured angle of external rotation (*P* = 0.04).

A decomposition of the interaction showed that external rotation decreased at 0° of hip flexion and increased as the hip flexion angle increased. The only significant difference found corresponded to the two triangles technique at 15° flexion (mean difference compared to the non-repaired state = 2.8° ± 3.8° or 8.8% increase in external rotation; *P* = 0.03).

**Conclusions:**

Different techniques of capsular plication result in a non-significant increase in hip external rotation when compared to unrepaired capsulotomies. Therefore, special attention should be paid at the time of capsular plication, which could be disadvantageous when done overzealously aiming to increase postoperative stability.

## Introduction

Hip instability has been identified by many authors as a cause of hip pain and result of injury in both the general population (Bedi et al., 2011; Sansone et al., 2012; Taber, 2011) and in athletic patients (Austin et al., 2014). It corresponds to “micro-motion” of the femoral head relative to the acetabular center (Taber, 2011). Whether caused by repetitive trauma in abduction and external rotation, generalised ligamentous laxity, iatrogenic causes, connective tissue disorders or a combination thereof (Blakey et al., 2018; Bowman et al., 2010; Philippon, 2002), instability induces an overload of the connective tissues which leads to hip discomfort, pain, and possibly osteoarthritis. In its normal state, the hip is an inherently stable articulation due to its “ball-and-socket joint” congruency (Bowman et al., 2010; Domb et al., 2013; Slikker et al., 2012). The articulating surface of the half-spherical acetabulum is augmented by a fibrocartilage (labrum) which creates a “suction effect” by obstructing the fluid flow in and out of the joint, thus enhancing stability (Bowman et al., 2010; Crawford et al., 2007; Domb et al., 2013; Nepple et al., 2014). The stability of the hip joint is also dependent on the mechanical properties (e.g. stiffness) of the joint capsule, which is composed of three thickened ligaments, and of the surrounding muscles (Bedi et al., 2011; Flack et al., 2011; Martin et al., 2008; Philippon et al., 2014).

Hip instability has been documented as a consequence of surgical intervention by several studies (Ranawat et al., 2009; Sansone et al., 2012; Wylie et al., 2016; Yeung et al., 2016) (Table 1). Based on evidence implying that any capsular opening will lead to some degree of joint instability or pain if not properly closed after surgery, most studies have argued for the potential advantages of performing capsular repair after hip arthroscopy (Austin et al., 2014; Bayne et al., 2014; Bedi et al., 2011; Domb et al., 2013; Harris et al., 2013a; Slikker et al., 2012). Hip instability, or micro-instability as termed by others is an ill-defined pathology. In the absence of dysplasic bone morphology, its diagnosis remains a significant clinical challenge (Bedi et al., 2011; Taber, 2011) and the need for capsular plication in such cases is still debated (Hebert1 et al., 2014; Chile et al., 2013). For better assessment of this problem, the effects of different capsular plication techniques (Bedi et al., 2011; Domb et al., 2013; Smith & Sekiya, 2010) on the mobility of the hip joint in a clinically relevant setting needs to be addressed.

**Table 1:** Studies on ranges of motion associated with hip capsular laxity from arthroscopy and imaging technique for diagnosis

Study	Diagnosis criteria for capsular laxity	Technique used	Sample size	Follow up	
				Assessment type	Time to follow up
M. Belemmi et al. 2014 (Chile et al., 2013)	Capsulotomy for FAI^a^	No closure	Not mentioned in abstract	Modified Harris Hip score Vail score WOMAC score	3 & 6 months
		total closure (capsulorrhaphy)			
J. Wylie et al. 2013 (Wylie et al., 2013)	Capsulotomy for FAI^a^ (patients chosen because of symptoms of instability after FAI surgery)	No closure	13 patients, 14 hips (from 324 patients)	Modified Harris Hip score Hip outcome score	Minimum follow up of 6 months
		Arthroscopic capsular repair			
C.T. Hebert et al. 2014 (Hebert et al., 2014)	Capsulotomy in cadaveric hips	No closure (intraportal capsulotomy)	10 hips, 5 left, 5 right (range: 28–82 years old)	Rotational force while flexed (90 degrees) and while extended (full) (before & after surgery)	None
		Capsulorrhaphy: (intraportal capsulotomy)			
		Capsulorrhaphy: (T capsulotomy)			
Bayne et al. 2014	Capsulotomy in cadaveric hips	Intact capsule	13 hips	External rotation torque while in neutral flexion & maximal flexion	None
		Transverse capsulotomy			
Magerkurth et al. 2013 (Magerkurth et al., 2013)	Retrospective assessment of imaging after laxity diagnosis at surgery	Magnetic resonance imaging	27 patients, 17 positive, 10 negative for hip joint laxity	Measurements of capsular & zona orbicularis thickness	None
Blakey et al. 2010 (Blakey et al., 2018)	Excessive external rotation at rest + pain	Dynamic MRI	11 hips (10 patients, averaged 31 years old, range 21–47)	Physical exam (FABER test, FADIR test, recoil test…) Beighton hypermobility score	None

^a^femoroacetabular impingement

This study aims to assess the ranges of motion associated with different hip joint capsular plications. More specifically, our research question concerned whether hip capsular plication would have an effect on limiting external rotation of the hip in extension when compared to leaving a mini-capsulotomy performed during hip arthroscopy untouched. We hypothesised that hip external rotation would be reduced with capsular plication and that the extent of this limitation would translate into a proportional impact on hip joint stability.

## Materials and methods

### Cadaver preparation

Six cadavers (5 men, 1 women) with an average age of 67 years (range, 56–74 years), all fresh frozen and stored at − 5 °C, were selected for the study. Previously unprepared consecutive cadaveric specimens were chosen in order to maintain the effect of all surrounding tissues around the hip while testing. C-arm fluoroscopy was used to excluded cadavers with pistol grip or cam deformity, acetabular retroversion (ischial spine sign and cross-over sign), acetabular dysplasia (center edge angle < 20°), Legg-Calvé-Perthes disease, slipped capital femoral epiphysis and history of hip fracture or surgery on plain radiographs.

### Experimental protocol

Following a thawing protocol at room temperature for 48 h, cadavers were kept at 4 °C for 36 h before manipulations. Every hip joint was examined for limitations in free range of motion in flexion, internal and external rotation prior to testing. Fluoroscopic frontal pelvic view evaluations were performed for every cadaver.

**Fig. 1:**
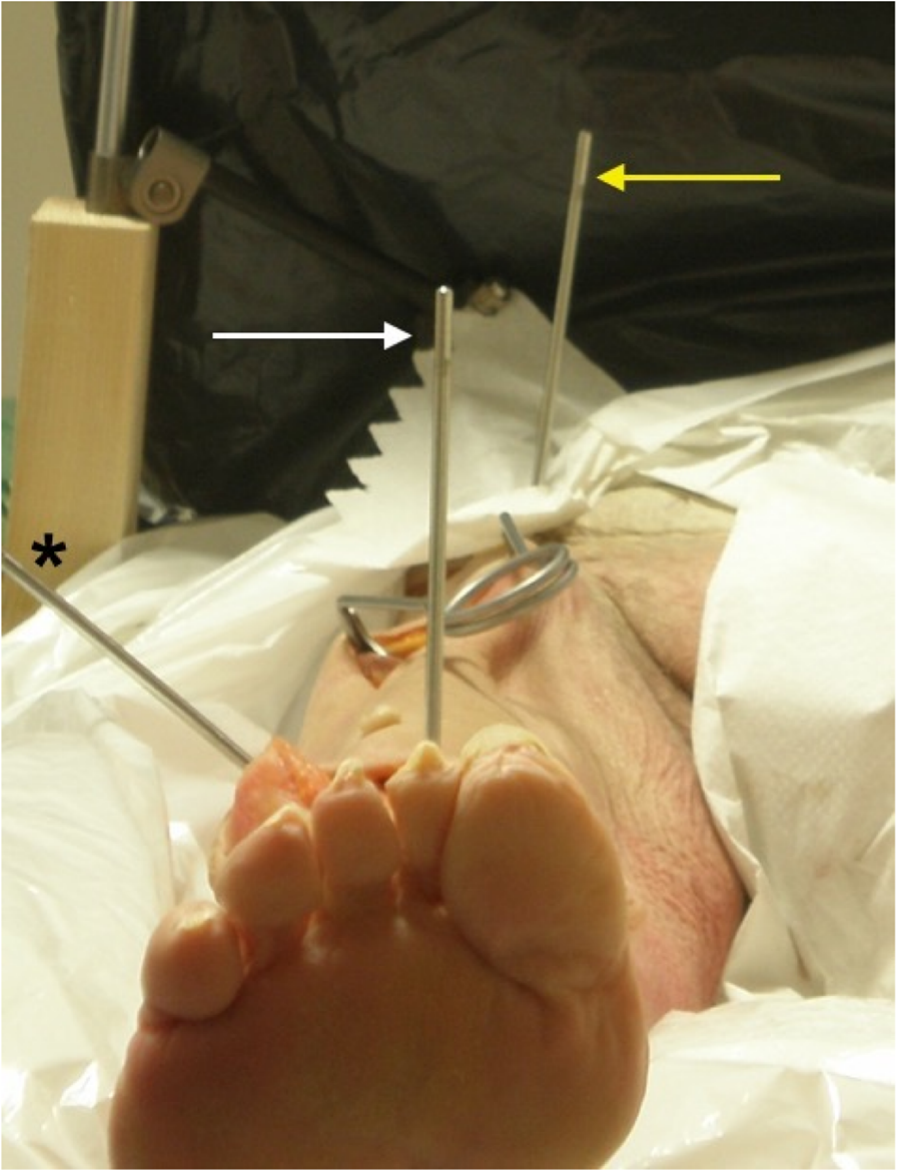
Photograph of the experimental apparatus with the camera used for data acquisition at the foot of the bed. Yellow arrow = ASIS reference pin; white arrow = reading pin in femur; * = lever pin

Upon beginning the surgical procedure, the whole cadaver was fixed to the operating room table using a custom made apparatus stabilized with four clamps. A 5 mm Steinman pin was inserted into each iliac wing and attached to the apparatus by means of connectors and 10 mm carbon rods from an external fixator. We then proceeded with the insertion of one 5 mm Steinman pin vertically (perpendicular to the leveled table) in the anterior superior iliac spine (pin set as reference for rotation) and a second pin, parallel to the first pin from anterior to posterior, in the supracondylar metaphysis of the distal femur (reading pin) 2 cm proximal to the femoral inter-epicondylar axis. These pins were vertically aligned to allow the measurement of the external rotation of the hip during manipulations. Another 5 mm Steinman pin was subsequently inserted laterally in the distal femur at 10 cm proximal to the lateral femoral epicondyle to serve as a lever to induce external rotation in the hip (Fig. 1).

In our study design, three capsular plication techniques were successively performed on each hip. For the intact hip and every subsequent plication, rotational torque was applied manually until a firm end-feel was felt on the previously described lever pin by a single operator (ELB). The operators were not blinded to the plication technique used due to the increasingly invasive order of these techniques. They thus logically had to be performed in the same order in every hip. With each plication technique, maximal external rotation of the hip was measured at 0°, 15° and 30° of hip flexion, as positionned with a goniometer. For every condition, 3 consecutive mesurements were made at each flexion angles before the next plication technique was applied.

### Surgical techniques

**Fig. 2:**
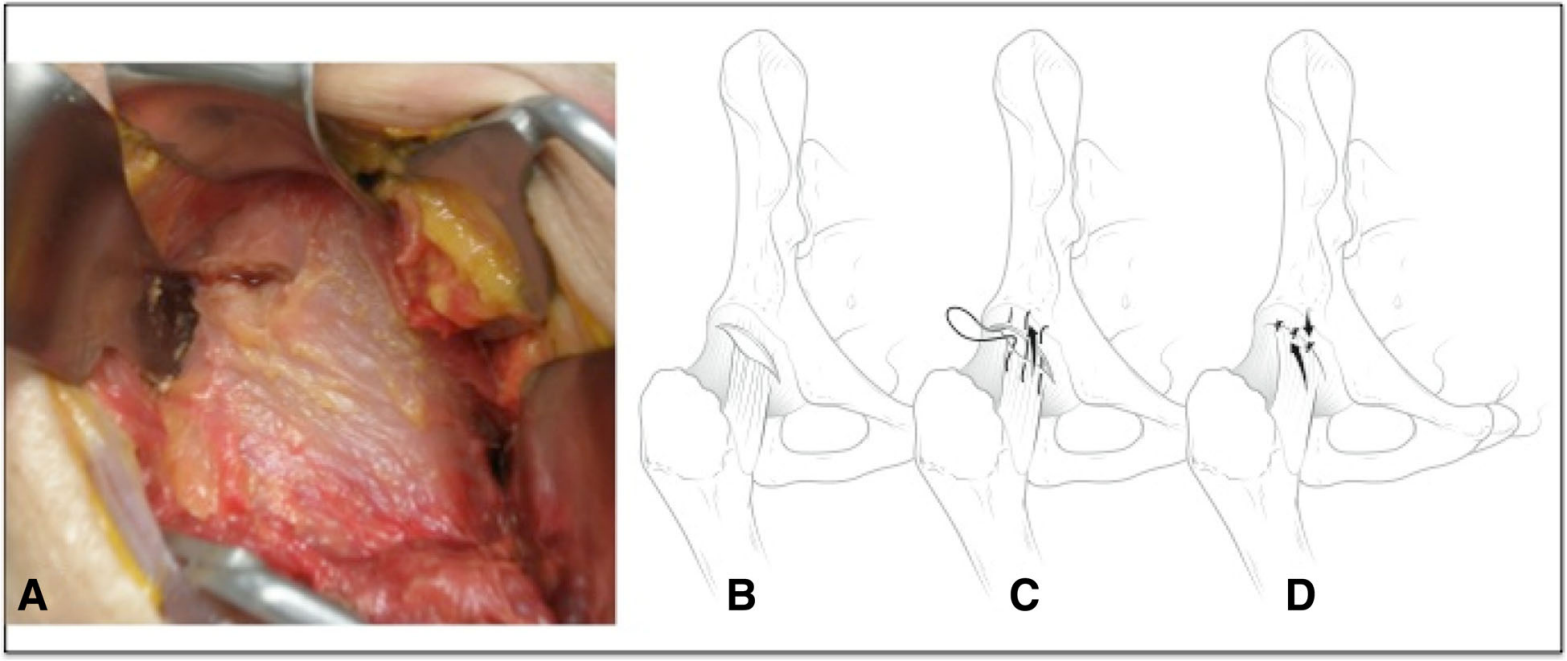
**a** and **b** shows the extent of the interportal capsulotomy performed initially. **c** demonstrates the primary plication technique with a slight shift of the distal limb from medial to lateral. **d** represents the end product of capsular closure which was completed with 3 sutures

A standard antero-lateral approach to the hip (Bertin & R ttinger, 2004) in the interval between tensor fascia lata and gluteus minimus, was performed on all hips with a special attention to protect the abductor muscles during the dissection. Special care was directed at preserving the capsule fibers and ligaments. The reflected head of the rectus was carefully elevated to allow for full surgical access to the anterior capsule. A 3 cm transverse (horizontal) capsulotomy was performed 1 cm distal from the origin of the capsule fibers on the antero-lateral acetabulum as to simulate the anterior to lateral, portal-to-portal, arthrotomy commonly used during hip arthroscopy (Fig. 2a and b) (Bedi et al., 2011; Domb et al., 2015; Harris et al., 2013b; Slikker et al., 2012).

**Fig. 3:**
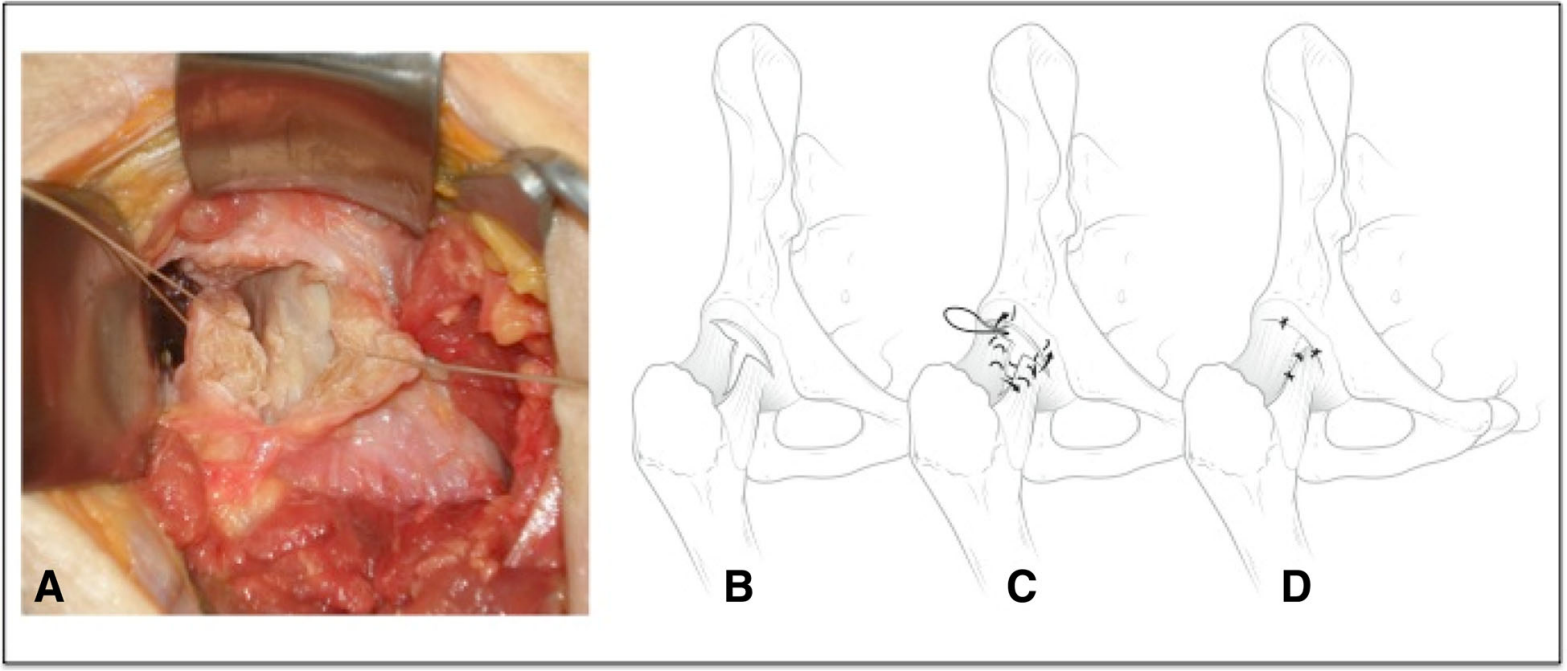
**a** and **b** demonstrate the extended limb of the capsulotomy as in a “T” with the division of the iliofemoral ligament between its medial and lateral arm. **c** represents the second plication with an attempt at imbricating both arms of the iliofemoral ligament before closing the interportal portion of the capsulotomy. **d** represents the end product of capsular closure which was completed with 3 sutures

**Fig. 4:**
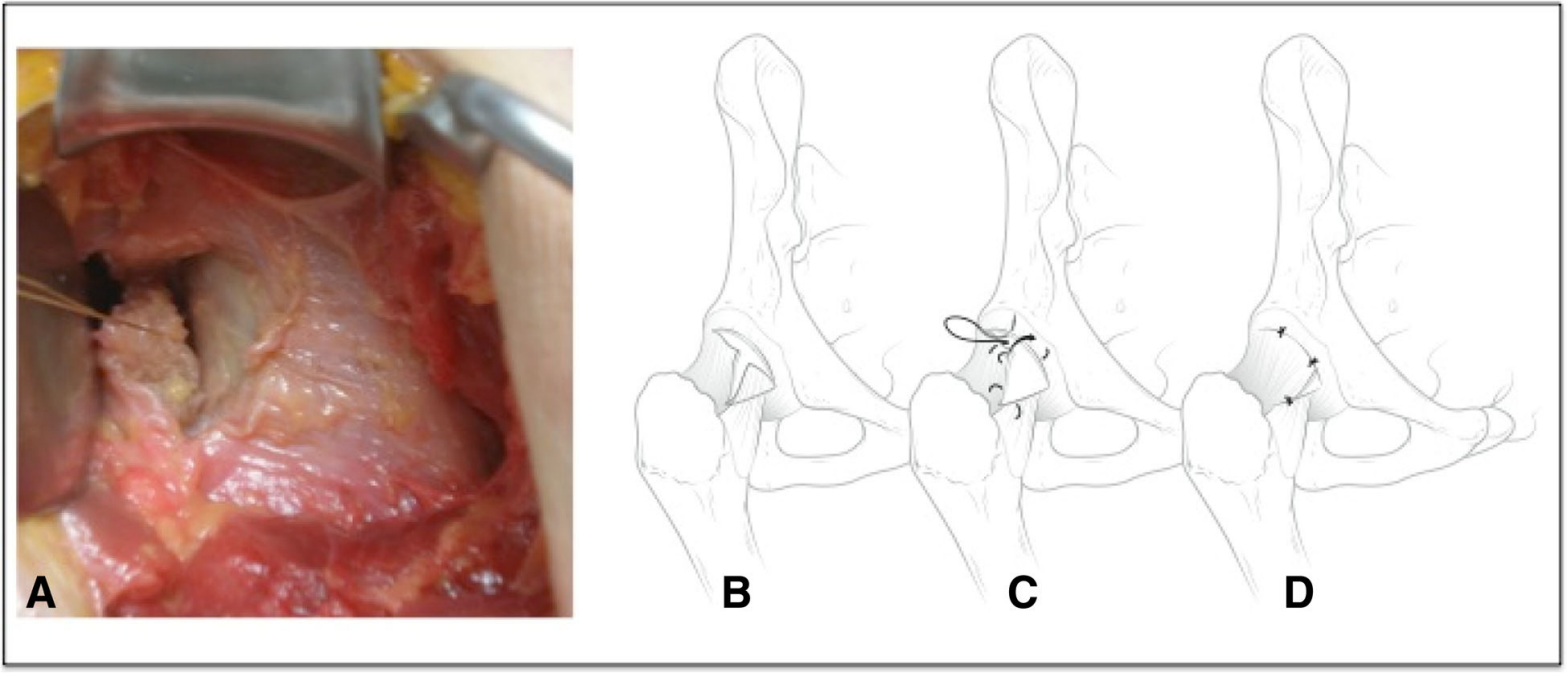
**a** and **b** demonstrates removal of the most inferior triangular flap of capsule. **c** represents the third plication technique, which brings the superior triangular portion of capsule down and towards the most medio-inferior portion of the interportal capsulotomy. **d** represents the end product of capsular closure which was completed with 3 sutures

The first plication technique consisted of a primary plication shift of the antero-lateral capsule. The distal-medial arm of the iliofemoral ligament was shifted toward the proximal-lateral arm to have a translation shift of approximately 5 mm and tightened using 1–0 resorbable sutures (Domb et al., 2013) so to strengthen the anterior capsule (Fig. 2c and d). The result was a slight shift of the distal limb from medial to lateral. No further capsular incision was made and the sutures were removed after measurements. The second plication technique consisted in adding a longitudinal arm to the capsulotomy, between the lateral arm and the medial arm of the iliofemoral ligament, to create a T-shaped capsulotomy (Fig. 3a and b). The resulting two triangular capsular flaps were overlaid onto each other by approximately 5 mm, plicated fully and tighly sutured (Bedi et al., 2011; Smith & Sekiya, 2010) with three 1–0 sutures in a double-breast manner (Fig. 3c and d). Again, once external rotation measurements were obtained, the sutures were removed. The third plication technique, called redrapping, consisted in excising the inferior capsular triangular flap (previously made in the second technique), and suturing the latero-anterior superior capsular flap to the medial arm of the iliofemoral ligament with three 1–0 sutures, superimposing the capsular edges for closure (see Fig. 4).

### Angular measurements

Using a 14′ stainless steel goniometer (Fabrication Enterprises Inc., White Plains, NY, USA), markers were positioned on the operating room table to mark the cadaveric leg position in the neutral position 0°, at 15° and at 30° of hip flexion. For each surgical plication technique, the measurements of the external rotation of the hip at 0°, 15° and 30° of flexion were obtained. The angle of external rotation was defined as the angle between the reference pin and the reading pin as seen in the transverse plane. The first dataset acquired on each hips represented the non-repaired state of the capsule after simulating the hip arthroscopy portal-to-portal capsulotomy. This measure served as the baseline external rotation for further comparison.

To standardize data acquisition, three measurements were made for each hip position and results were averaged. The measurement of the external rotation angle was carried out using a tripod mounted digital camera positioned perpendicular to the transverse plane. Each photograph was labelled according to surgical technique, hip position and trial number. Using an image processing and analysis software (ImageJ, https://imagej.nih.gov/ij/, 1997–2014), external rotation was measured from the digital photographs and compiled for each surgical technique and hip flexion angle.

### Statistical analysis

Two analyses were performed: one to evaluate whether each plication technique affects the degree of external rotation measured at the hip; and another to evaluate whether the impact of plication differed depending on hip flexion. External rotation data were initially analyzed by repeated measures two-way analyses of variance (ANOVAs) and, when necessary, a Student’s T-test was conducted for each variation in external rotation angle (post plication – pre plication) to determine if change in external rotation was significantly different from 0. Statistical significance was set at an α value of 0.05. Statistical analysis was performed using STATISTICA 10.0 (StatSoft, Inc., Tulsa, OK, USA).

## Results

Six fresh frozen undisected cadavers were studied (5 men, 1 women) for a total of 12 hips tested. In the non repaired state, external rotation averaged 26.3° ± 7.5°at 0° of flexion, 29.1° ± 5.8 at 15° of flexion and 31.1° ± 7.9 at 30° of flexion. With the first plication technique (primary plication shift), external rotation averaged 24.9° ± 7.9°at 0° of flexion, 30.3° ± 6.8 at 15° of flexion and 34.0° ± 8.9 at 30° of flexion. With the second plication technique (two triangle technique), external rotation averaged 26.1° ± 8.4°at 0° of flexion, 31.9° ± 8.2 at 15° of flexion and 33.3° ± 9.5 at 30° of flexion.With the third plication technique (redrapping), external rotation averaged 25.8° ± 8.6°at 0° of flexion, 30.9° ± 7.3 at 15° of flexion and 32.0° ± 9.1 at 30° of flexion.

**Fig. 5:**
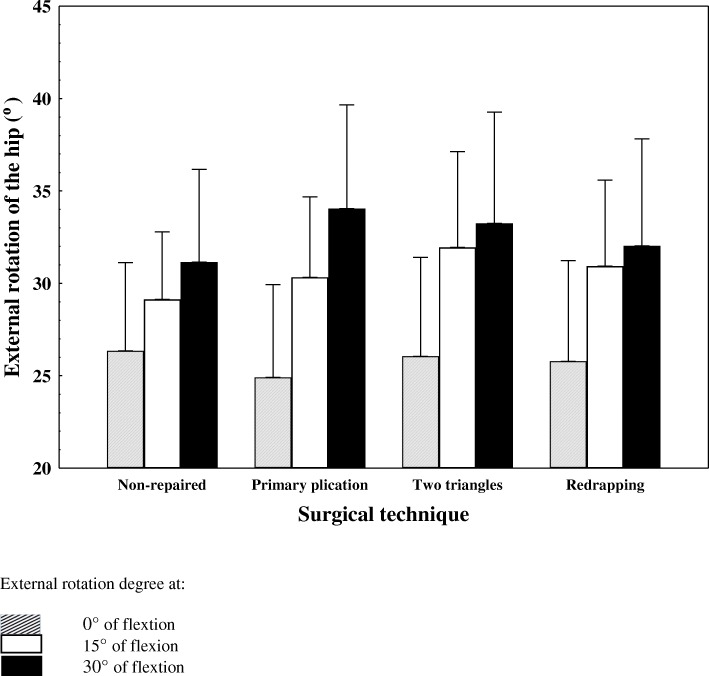
Mean external rotation of the hip for each surgical technique. Vertical bars denote 95% confidence intervals

A significant relationship was found between Plication Technique and Angle of flexion factors for the measured angle of external rotation (*P* = 0.04; Fig. 5) (suggesting that either the technique used, the flexion angle or both these factors had an effect on external rotation.). A decomposition of the interaction showed that the general tendency for external rotation was to be reduced while at 0° of hip flexion and to be increased at every other flexion angle for every different plication. The only significant difference found corresponds to the second technique (two triangles) at 15° of hip flexion (mean difference compared with the non repaired state = 2.8° ± 3.8° or 8.8% increase in external rotation; *P* = 0.03; Table 2).

**Table 2:** Mean variation in angle of external rotation (post plication – pre-plication)

	TECHNIQUE USED								
	FIRST TECHNIQUE Primary plication			SECOND TECHNIQUE Two triangles			THIRD TECHNIQUE Re-draping		
	Hip flexion angle (°)			Hip flexion angle (°)			Hip flexion angle (°)		
SAMPLES	0.0	15.0	30.0	0.0	15.0	30.0	0.0	15.0	30.0
A	−4.1	3.8	11.3	−1.0	5.1	12.9	1.3	6.5	12.7
B	1.3	2.7	20.4	2.9	10.8	23.0	3.4	3.2	14.5
C	0.6	0.3	−3.7	−2.6	0.2	−3.3	−4.5	−0.9	−6.9
D	−2.4	1.9	−6.0	5.5	5.4	− 3.5	2.4	2.6	− 0.1
E	−4.4	−5.7	− 0.4	−3.2	0.8	−2.2	−6.5	− 0.4	− 2.4
F	−7.0	−3.0	−0.4	−3.7	−1.9	− 3.2	− 5.2	3.3	− 4.7
G	0.5	5.0	4.3	1.1	1.8	−1.3	2.2	6.0	1.6
H	0.6	2.2	1.5	0.1	3.4	−0.1	3.2	1.2	−4.0
I	0.6	1.9	2.9	−0.4	2.2	0.3	−2.3	0.3	−3.3
J	−1.9	− 2.0	1.2	− 3.6	− 3.4	− 1.0	− 2.4	−4.1	0.4
K	− 1.0	1.8	3.7	−0.2	3.4	6.5	−0.2	0.0	4.1
L	0.1	5.4	−0.2	1.6	5.8	−3.0	1.8	3.9	−1.3
MEAN	−1.4	1.2	2.9	−0.3	2.8	2.1	−0.6	1.8	0.9
STANDARD DEVIATION	2.6	3.3	7.0	2.8	3.8	8.2	3.5	3.1	6.7
*P* value (compared with no variation)	0.08	0.23	0.18	0.72	0.03	0.39	0.59	0.07	0.66

## Discussion

Results of the current study, even if not statistically significant, showed that capsular plication tended to increase external rotation for different hip flexion angles. Quite the opposite of what was previously proposed (Austin et al., 2014; Bayne et al., 2014; Bedi et al., 2011; Domb et al., 2013; Harris et al., 2013a; Slikker et al., 2012), this suggests that capsular plication may be somewhat counterproductive or at best, have a neutral effect when trying to increase stability in the hip joint post-operatively and thus, that it should be completed with an adjusted level of expectation.

Some studies examining hip range of motion in cadaveric specimens deemed it pertinent to standardize the rotational forces applied on the hips by having an apparatus apply a constant force for each trial (Abrams et al., 2015; Bayne et al., 2014; Hebert1 et al., 2014). However, authors commonly disagree on the proper amount of force to be applied in order to reproduce in vivo conditions. Forces ranging from 0.588 NM (Bayne et al., 2014) to 10 NM have been tested. Despite this, some authors conclude that capsular plication would be beneficial in restoring pre-operative ranges of motion (Bayne et al., 2014) or at the very least, that capsulotomy increases ranges of motion compared to the normal hip and that judicious capsular management is indicated during arthroscopic procedures (Abrams et al., 2015). Other studies still debate the benefits of capsular repairs in treating hip instability (Hebert1 et al., 2014; Chile et al., 2013). Similarly to the current study, the previously stated studies used external rotation of the hip as a way to measure hip instability. On the other hand, these same studies have worked on heavily dissected specimen, leaving only bone, capsule and ligaments. Removal of soft tissue surrounding the hip joint, which are key factors in dynamic and passive hip stability in the living individual (Dwyer et al., 2014), may in fact diminish the clinical relevance of these studies by rendering the hip more vulnerable at extreme ranges of motion. Whereas a cadaveric capsular plication model may not represent a perfect clinical application, it represents the first step necessary to demonstrate a change in hip external rotation limitation. Further biomechanical investigations will continue to elucidate this concept.

This study has some limitations. The study was conducted on cadavers using surgical knives and not a diathermy probe as happens in arthroscopy. This is a study limitation because the use of the diathermy probe to open the capsule causes a shrinkage of the capsulotomy edges. In turn this will lead to a tighter closure. As a result, a limited range of motion might occur. Another study limitation is the location of arthroscopic capsulotomy that might differ a few millimetres from open capsulotomy. A small sample size may have limited our ability to reach statistical significance should it truly exist. It is possible that a larger sample size may have been able to show a more clinically significant difference between capsular plication techniques. However, care was taken to ensure reproducibility and accuracy of results with triplicate and digital testing. Furthermore, there are always limitations in cadaveric studies as the dynamic soft tissue envelope (muscles) and its contributions are not captured (Dwyer et al., 2014). Despite this, in this study, care was taken to preserve all muscle attachments to mimic an in vivo setting as best as possible. The torque applied in external rotation onto the lever pin was not standardized, although it was done by the same operator, but it was through manipulation. Although a few hip flexion positions were tested, these angles enabled ease of cadaveric preparation, testing, and measurements. Further, they were chosen as they are representative of common everyday hip positions while standing and walking (Anderson & Pandy, 2001; Lewis et al., 2010). More comprehensive flexion angle testing would require a more sophisticated methodology but may also provide a more global understanding of the role of capsular plication.

The study design made it difficult to solely assess the effect of one capsular closure technique without having potential remnant effects from the prior techniques. Alternative sequence of execution of the different techniques may have represented a better methodological design but this was not possible due to the incisions to the native capsule required to complete each technique. Thus, they were performed in an increasingly invasive order. Finally, our surgical model and the results of testing may not be generalizable to patients with hip deformity, hyperlaxity, hypermobility syndromes or connective tissue disorders or in cases of micro-instability (Bedi et al., 2011; Bowman et al., 2010; Harris et al., 2013b; Taber, 2011). It is possible that using the plication techniques in already hyperlax joint capsules may lead to more significant results from capsular plication. Therefore, further work on the development of a distended capsular model is warranted.

## Conclusions

We found that every plication technique used in our study tended to show a non significant increase in external rotation, compared to the unrepaired capsulotomy state. This result therefore suggests that capsular plication of the hip may not be the only factor to address when treating instability of the non deformity hip joint.
